# Chitosan Gel Sheet Containing Polymeric Micelles: Synthesis and Gelation Properties of PEG-Grafted Chitosan

**DOI:** 10.3390/ma10091075

**Published:** 2017-09-13

**Authors:** Chikara Yoshida, Yusuke Uchida, Tomoki Ito, Taku Takami, Yoshihiko Murakami

**Affiliations:** Department of Organic and Polymer Materials Chemistry, Faculty of Engineering, Tokyo University of Agriculture and Technology, 2-24-16 Naka-cho, Koganei, Tokyo 184-8588, Japan; c-yoshida@smclab.co.jp (C.Y.); yuchida2@mmm.com (Y.U.); s150662q@st.go.tuat.ac.jp (T.I.); taku.takami@riken.jp (T.T.)

**Keywords:** sheet, gel, chitosan, PEG-modification, polymeric micelle, wound-dressing

## Abstract

Wound-dressing sheet biomaterials can cover wound sites and enhance wound healing. In this study, a detailed evaluation of the factors affecting both the PEG modification percentage (PMP) in poly(ethylene glycol) (PEG)-grafted chitosan synthesis and the gelation properties of PEG-grafted chitosan was presented for constructing our novel hybrid hydrogel sheet consisting of PEG-grafted chitosan (a gel-forming polymer) and a reactive polymeric micelle (a crosslinker). It was confirmed that various factors (i.e., the weight ratio of PEG/chitosan, the pH of the buffer solution, reaction times, and reaction temperatures) in the preparation stage of PEG-grafted chitosans affected the PMP of PEG-grafted chitosans. Furthermore, the PMP of PEG-grafted chitosans affected their gelation properties. Finally, a ‘flexible’ hydrogel sheet that can be reversibly dried and moistened was successfully obtained. The dried rigid, thin sheet is expected to be suitable for stable preservation. The results obtained in this paper show that the incorporation of drug carriers into biomaterials is a novel approach to improve functionality.

## 1. Introduction

Conventional dry wound-dressing materials (such as gauze) often hinder wound healing by absorbing exudates, including macrophages and growth factors. To overcome the defect, “hydrogel-based” wound-dressing materials have been used to protect wound sites from drying out, prevent microbial invasion, and maintain the activity of growth factors [1,2].

The controlled sustained release of compounds from hydrogels is difficult because the release mechanism depends mainly on two phenomena: the degradation of polymeric networks and the diffusion of compounds through the hydrogel medium. In these regards, we have developed a novel ‘hybrid’ approach: the incorporation of functional block copolymers and/or their self-assembly (polymeric micelles) into base materials (such as gel [3,4,5,6,7], sheets [8,9,10], and particles [11,12,13,14,15]) for the construction of biomaterials for drug delivery systems. Polymeric micelles have a core that can incorporate either hydrophobic [16,17,18,19,20,21] or hydrophilic [22,23] compounds and release drugs by means of either the dilution-induced collapse or the degradation of micelle-forming polymers. Thus, the hybrid material design approach (i.e., the incorporation of micelles into hydrogels) is expected to help give the hydrogels various controllable drug release properties.

Chitosan is a natural polysaccharide that is obtained by deacetylating chitin, a main component of the exoskeleton of crustaceans. Because of its potentially beneficial properties such as biodegradability, non-toxicity, and antimicrobial activity [24], chitosan has been used in many biomedical and pharmaceutical applications, including the wound-healing process [25,26]. However, strong intermolecular hydrogen bonds on chitosan backbones make the chitosan rigid, insoluble, and resistant to the construction of ‘hydrogel-based’ chitosan biomaterials. To address these problems, a polymeric modification is one of the most effective approaches to improve chitosan’s hydrophilic characteristics [27,28]. Poly(ethylene glycol) (PEG) is a highly hydrophilic polymer that has served as a non-inflammatory and non-immunogenic modifier for proteins and drugs. The polymers and materials modified with PEG were found to be less thrombogenic due to the flexible backbone and hydrophilicity of PEG [29,30,31,32,33,34,35].

**Figure 1:**
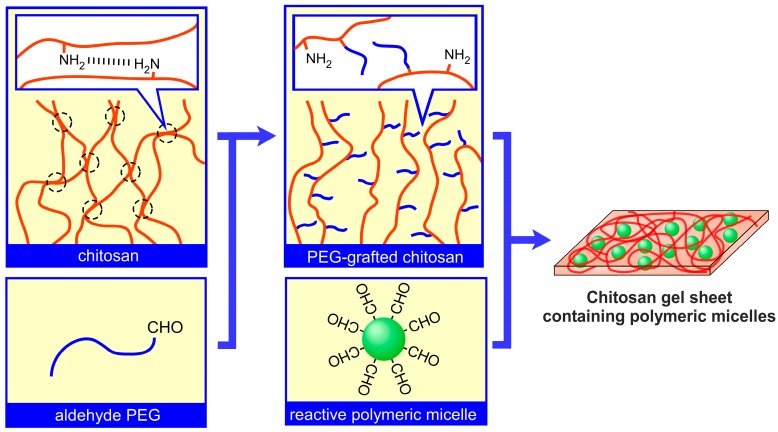
Hydrogel sheet consisting of PEG-grafted chitosan and crosslinkable polymeric micelles.

We have proposed a preliminary design for a novel transparent PEG-grafted chitosan-polymeric micelle hybrid gel sheet [36]. Since the visual observation of the healing process is important, transparent would dressings have been an attractive development object [37]. The hydrogel consists of a poly(ethylene glycol) (PEG)-grafted chitosan (PEG-grafted chitosan) and a crosslinkable polymeric micelle (Figure 1). The preparation of PEG-grafted chitosan serves to increase the solubility and improve the biocompatibility of the chitosan. Although there have been numerous studies on PEG-grafted chitosan and its usage in biomaterials, the characteristic feature of our PEG-grafted chitosan-polymeric micelle hybrid gel sheet is that only polymeric micelles were used as crosslinkers for gelation. It is expected that this structural feature gives the sheet controllable drug-release properties. However, two problems remain to be addressed in the preliminary study: the factors affecting both the PEG modification percentage (PMP) of chitosan and the gelation properties of chitosan hydrogels are still unclear, and the flexibility of finally obtained ‘sheets’ is lower than expected. Thus, in the present paper, we directly address these two problems by presenting a detailed evaluation of the factors affecting both the PMP in PEG-grafted chitosan synthesis and the gelation properties of PEG-grafted chitosan. Finally, we discuss how we successfully prepared a flexible hydrogel sheet with reversible dry and wet forms.

## 2. Materials and Methods

### 2.1. Materials

Ethylene oxide (Sumitomo Seika Chemicals Co., Osaka, Japan) was purified by distillation in the presence of CaH_2_. DL-Lactide (Tokyo Chemical Industry Co., Tokyo, Japan) was recrystallized twice from ethyl acetate. Under reduced pressure, 3,3-diethoxypropanol was distilled with sodium. Potassium naphthalene was synthesized through the mixing of potassium and naphthalene in anhydrous tetrahydrofuran (THF) for 18 h. Chitosan (with a degree of deacetylation of more than 98%, and degree of viscosity between 40 and 100 mPa s) was purchased from Dainichiseika Color & Chemicals Mfg. Co., Ltd. (Tokyo, Japan). All other reagents were of analytical grade and were used without further purification.

### 2.2. Synthesis and Characterization of PEG Derivatives

**Figure 2:**
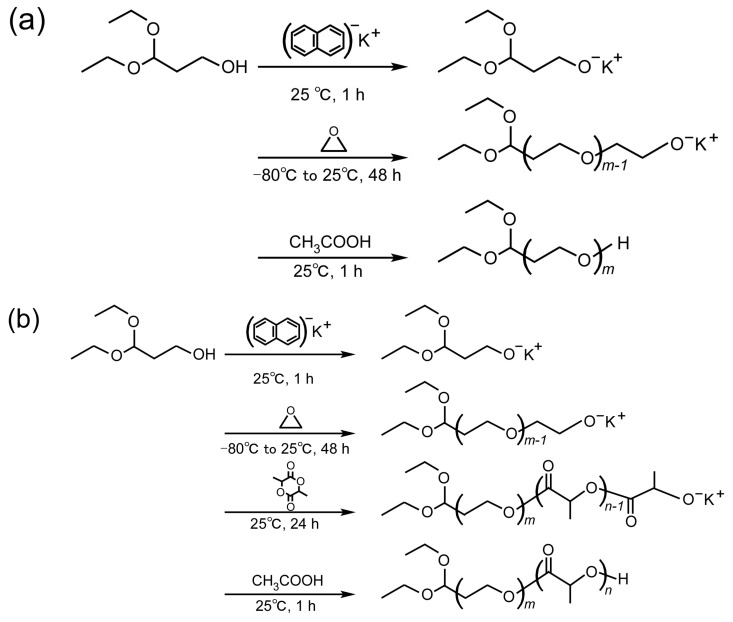

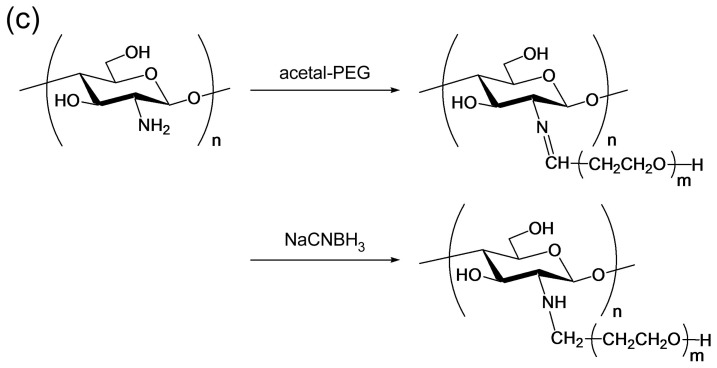
Scheme of the synthesis of (**a**) acetal-PEG; (**b**) acetal-PEG-*b*-PLA; and (**c**) PEG-grafted chitosan.

Acetal-terminated PEG (abbreviated as acetal-PEG), a modifier of chitosan, was synthesized by means of ring-opening polymerization of ethylene oxide in anhydrous THF (Figure 2a). 3,3-Diethoxypropanol (1.5 mmol) and potassium naphthalene (1.5 mmol) were mixed in THF for 1 h. The purified ethylene oxide (195 mmol) was then added to the obtained potassium 3,3-diethoxypropioxide solution (40 mL), and polymerization was carried out for 48 h at 25 °C. The resulting polymer was precipitated into diethyl ether, filtrated, and lyophilized in benzene.

The acetal-terminated PEG-*block*-poly(lactic acid) block copolymer (abbreviated as acetal-PEG-*b*-PLA), a micelle-forming polymer, was synthesized by means of ring-opening polymerization of both ethylene oxide and DL-lactide in anhydrous THF (Figure 2b). 3,3-Diethoxypropanol (3 mmol) and potassium naphthalene (3 mmol) were mixed in THF for 1 h. The purified ethylene oxide (153 mmol) was then added to the obtained potassium 3,3-diethoxypropioxide solution (40 mL) and polymerization was carried out for 48 h at 25 °C. After polymerization, the purified DL-lactide (79.3 mmol) was added to the solution. The resulting polymer was precipitated into cold 2-propanol, stored in a freezer overnight, centrifuged at 10,500 rpm for 15 min, and lyophilized in benzene.

The molecular weights of the obtained polymers were determined based on gel permeation chromatography (GPC) (column: TSKgel G3000H_HR_, TOSOH, Tokyo, Japan; eluent: *N*,*N*-dimethylacetamide (DMF) in the presence of 10 mM LiBr; flow: 1 mL/min; column temperature: 40 °C) and ^1^H NMR (equipment: AL-300, 300 MHz, JEOL Ltd., Tokyo, Japan; solvent: deuterium chloroform).

### 2.3. Synthesis and Characterization of PEG-Grafted Chitosans

Chitosan was dissolved in a 0.1 M acetic buffer solution whose pH was 3.6–5.6. Acetal-PEG that had been dissolved in an acetic buffer solution was added to the chitosan solution and stirred for 3–15 h at 3–60 °C (Figure 2c). The weight ratio of acetal-PEG and chitosan (PEG/chitosan) varied from 5 to 25. NaCNBH_3_ in an acetic buffer solution was then added to the reaction solution and stirred for 18 h at room temperature. For the removal of the unreacted PEG, the resulting reaction solution was freeze-dried, redispersed to acetone, and centrifuged at 15,000 rpm for 15 min. The resulting precipitate was dissolved in an acetic buffer solution, dialyzed against water with a dialysis membrane (molecular weight cut-off: 10 kDa; Spectrum, Houston, TX, USA), and freeze-dried. Finally, the white powder obtained was characterized by ^1^H NMR, with deuterium oxide as a solvent.

### 2.4. Quantitative Analysis of PEG-Grafted Chitosans by Means of 2,4,6-Trinitrobenzene Sulfonic Acid (TNBS) Measurement

Basically, the PEG modification percentage (PMP) of the PEG-grafted chitosan was determined with 2,4,6-trinitrobenzene sulfonic acid (TNBS) by means of an analysis of the amount of free, unmodified amino groups, according to a previously reported method [38] with a slight modification. First, chitosan and PEG-grafted chitosans (whose concentrations were adjusted to be 2.5 mg/mL with a chitosan base) were dissolved in an acetic buffer solution, and a 0.36 w/v % TNBS solution was prepared in an acetic buffer solution. To 2 mL of the obtained chitosan or PEG-grafted chitosan solutions, 1 mL of the TNBS solution was added and stirred for 4 h at room temperature. TNBS reacts with unmodified amino groups and produces a yellow color. The extent of the color’s intensity depends upon the content of unmodified amino groups of PEG-grafted chitosan. The absorbance of the solutions was measured at 345 nm by means of an ultraviolet-visible spectrophotometer (V-630 bio, JASCO Co., Tokyo, Japan). The PEG modification ratio was calculated on the basis of a comparison between the absorbance of both the PEG-grafted chitosan and the chitosan.

### 2.5. Evaluation of the Solubility of PEG-Grafted Chitosans by Visual Observation and Static Light Scattering Measurement

The effect that the PMP had on the solubility of PEG-grafted chitosan was evaluated by means of static light scattering measurement with a Zetasizer Nano ZS (Malvern Instruments, Worcestershire, UK). First, chitosan and PEG-grafted chitosan were dissolved in water where the concentration of each component was varied from 0.4 to 1.0 g/L. A Debye plot was obtained from the Rayleigh equation, which featured the results of the static light scattering measurement in the range of the above concentration. The second virial coefficient was calculated from the slope of the Debye plot.

### 2.6. Preparation and Characterization of an Aldehyde-Terminated Polymeric Micelle Formed from Block Polymers

Acetal-PEG-*b*-PLA was dissolved in 5 mL of DMF. Then, the solution was dialyzed against water by means of a dialysis membrane (molecular weight cut-off: 1 kDa; Spectrum, Houston, TX, USA) for 24 h. HCl was added to the polymeric micelle solution so that the pH would adjust to 2. The solution was mixed for 2 h, thus converting the acetal group into an aldehyde group on the surface of the micelles. To stop the reaction, the pH of the solution was adjusted to 5 or 7 through the addition of an aqueous NaOH solution. The polymeric micelles having the aldehyde terminus on the surface were finally obtained through dialysis against water for 24 h, which enabled the removal of the salt. The size of the polymeric micelles was determined in water at 25 °C with dynamic light scattering measurement, made possible by the Zetasizer Nano-ZS.

### 2.7. Preparation and Characterization of an Aldehyde-Terminated Polymeric Micelle Formed from Block Polymers

To determine the hydrogel’s properties (storage modulus (G’), loss modulus (G”), and the gelation time), we used an RS600 rheometer (Thermo Fisher Scientific, Dreieich, Germany) at a gap of 1.0 mm and a shear stress of 1.0 Pa with a frequency of 1 Hz. The solutions containing either a PEG-grafted chitosan (0.1 mL; 0.1 w/w % with a chitosan base; pH 3–11 that was adjusted with HCl/NaOH) or the crosslinker (0.2 mL; 30 w/w %; pH 5 or 7 that was adjusted with HCl/NaOH) were introduced to the sample plate (15 mm in diameter) of the rheometer at 37 °C. For this purpose, we used a commercially available two-pronged needle that consisted of two syringes. Polymeric micelles served as crosslinkers in the formation of hydrogel. To prevent the hydrogel from drying, we covered the outside of the sample plate. Next, we prepared a chitosan hydrogel sheet by mixing the solutions containing either a PEG-grafted chitosan (1.1 w/w %, 66% of PEG modification percentage (PMP), 0.2 mL) or a polymeric micelle (30 w/w %, 0.3 mL). The mixed solution was introduced at a gap (1.0 mm) between two plates made of acrylic acid resin. After three days, the obtained sheet was immersed in water for 5 min. The transmittance of the film was determined at 600 nm by means of UV–vis spectrophotometer (V-630 bio, JASCO, Tokyo, Japan).

## 3. Results and Discussion 

### 3.1. Synthesis and Characterization of PEG Derivatives

**Figure 3:**
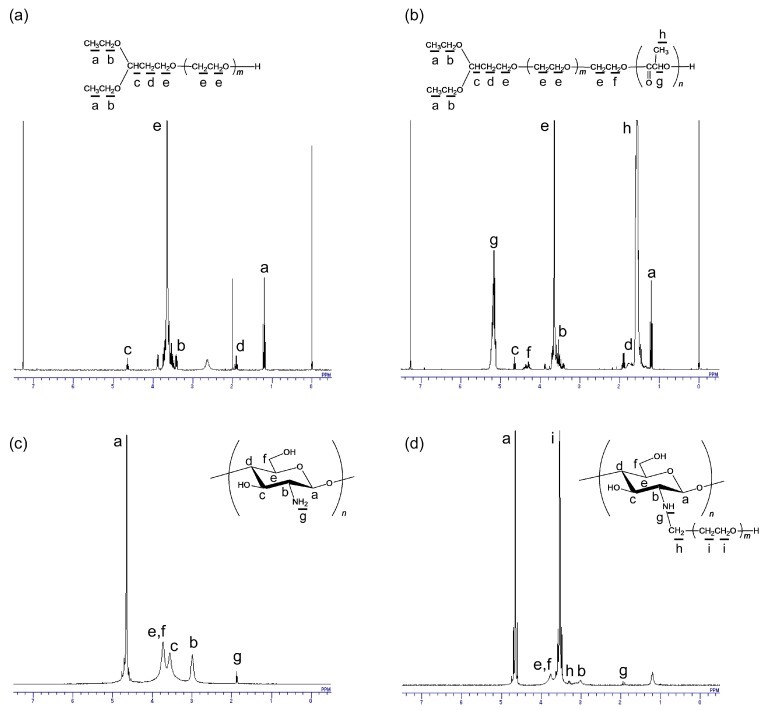
^1^H NMR spectra of (**a**) acetal-PEG; (**b**) acetal-PEG-*b*-PLA; (**c**) chitosan; and (**d**) PEG-grafted chitosan.

The obtained PEG derivatives, acetal-PEG and acetal-PEG-*b*-PLA, were characterized by ^1^H NMR and GPC. The ^1^H NMR spectra of the PEG derivatives shown in Figure 3a,b indicate successful synthesis of the PEG derivatives. The number-average molecular weight of the acetal-PEG was determined on the basis of GPC, whereas the number-average molecular weights of the acetal-PEG and the PLA blocks of the acetal-PEG-*b*-PLA were determined on the basis of GPC and ^1^H NMR, respectively. The respective number-average molecular weights of acetal-PEG and acetal-PEG-*b*-PLA were determined to be 4800 (*M*_w_/*M*_n_: 1.05) and 4700 (*M*_w_/*M*_n_: 1.20, with an acetal-PEG block (*M*_n_: 1800, *M*_w_/*M*_n_: 1.06) and PLA block (*M*_n_: 2900). ^1^H NMR also showed that the acetal groups were introduced to 85% and 82% of the termini of acetal-PEG and acetal-PEG-*b*-PLA, respectively. These obtained PEG derivatives were used for the subsequent experiments. Acetal-PEG was used as a modifier of chitosan because the aldehyde group (to which the acetal group had been converted) possessed the feature wherein acetal-PEG could bind with the amino group present on the chitosan backbone. Acetal-PEG-*b*-PLA served as a micelle-forming polymer to form aldehyde-terminated polymeric micelles acting as crosslinkers. The aldehyde-terminated polymeric micelles were used as crosslinkers because the aldehyde group on the surface of the polymeric micelle could bind with the amino groups of the chitosan backbone, i.e., the hydrogel was formed according to the Schiff base formation reaction. The diameter of the aldehyde-terminated polymeric micelles was determined to be 25 nm by means of a dynamic light scattering measurement.

### 3.2. Synthesis and Characterization of PEG-Grafted Chitosans

Figure 3c,d present the typical ^1^H NMR spectra of chitosan and PEG-grafted chitosan. In Figure 3d, the peaks corresponding to the signals of the methylene group of the PEG (H-i) and to the bond between chitosan and PEG (H-h) appeared at near 3.55 and 3.30 ppm, respectively, in the spectra of the PEG-grafted chitosan. These results indicate that the PEG chains were successfully introduced to the chitosan backbone, because the two peaks did not appear in the spectra of the native chitosan, as shown in Figure 3c.

Evaluation of PMP in the PEG-grafted chitosans was important for the design of chitosan-based hydrogels, because the increase in PMP results in a decrease in the reaction efficiency between PEG-grafted chitosans and aldehyde-terminated polymeric micelles. In our previous study, we reported that PMP increased proportionally to increase in the PEG-to-chitosan weight ratio in the synthesis condition of PEG-grafted chitosans [36], where one would determine PMP by comparing H-e,f (the monosaccharide residue of both the PEG-grafted chitosan and the chitosan) and H-h (the end of the methylene group of the PEG chain of PEG-grafted chitosan) in NMR spectra. However, two problems remain unresolved: (1) PMP determination based on NMR spectra yields significant errors in some cases (because the peak shape of H-e,f and H-h are affected by the peak shape of H-i under a high PEG concentration); and (2) although considerable research on PEG-grafted chitosans has been reported to date [39,40,41], it has been still difficult to clarify the main factors affecting PMP. In the present paper, we thus determined PMP on the basis of two analyses, a ^1^H NMR analysis and a 2,4,6-trinitrobenzene sulfonic acid (TNBS) reaction analysis.

**Figure 4:**
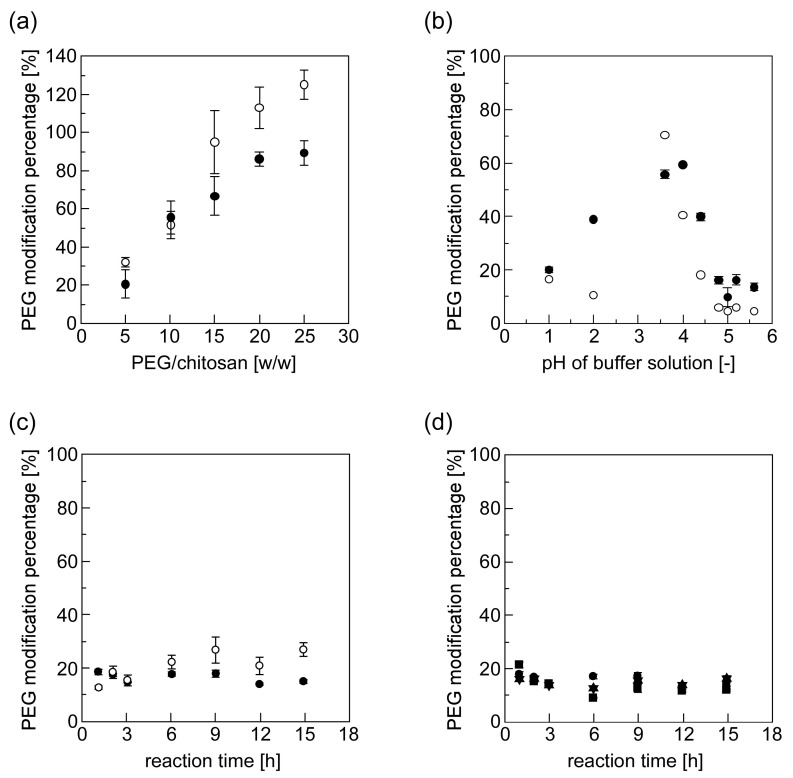
The effect of various factors in the preparation stage of PEG-grafted chitosans on their PMP by means of ^1^H NMR measurement (open circle) and TNBS assay (close circle). The factors evaluated were (**a**) the PEG-to-chitosan weight ratio; (**b**) the pH of the buffer solution; (**c**) a reaction time for PEG-chitosan conjugation; and (**d**) a reaction temperature (triangle: 60 °C, square: 37 °C, circle: 25 °C, and reverse triangle: 4 °C; the horizontal axis was the reaction time for the PEG-chitosan conjugation).

Figure 4 shows the effects that various factors (the weight ratio of PEG/chitosan, pH of buffer solution, reaction time, and reaction temperature) in the PEG-grafted chitosan preparatory stage can have on PEG-grafted chitosan’s PMP. First, we evaluated this PMP by using the ^1^H NMR spectra of the PEG-grafted chitosan (open circles in Figure 4). The PMP was evaluated in terms of the relative intensities between the peaks H-e,f and H-h, which are respective to the monosaccharide residue of the PEG-grafted chitosan and the end of the methylene group of the PEG chain of PEG-grafted chitosan. Figure 4a shows the effects that the weight ratio of PEG/chitosan in the PEG-grafted chitosan synthesis condition had on the PMP. The PMP increased proportionally to increases in the PEG-to-chitosan weight ratio, and these results are identical to the results in our previous paper [39]. However, a PEG-grafted chitosan’s PMP exceeding 100% was unexpectedly obtained when the PEG-to-chitosan weight ratio exceeded 20, because two peaks, corresponding respectively to H-e,f and H-h, in the ^1^H NMR spectra lost their accuracy and sharpness owing to the presence of a large peak corresponding to H-i under a high PEG concentration.

Then, we evaluated the PMP of PEG-grafted chitosan by using the TNBS assay (closed circles in Figure 4). Basically, TNBS-assay evaluation of this PMP enabled us to analyze the amount of free, unmodified amino groups [39]. Figure 4a clearly shows that PMP increased, and reached the saturation value (below 100%), as the PEG-to-chitosan weight ratio increased. Figure 4b shows the effect that the pH of the acetic acid buffer in the synthesis condition of the PEG-modified chitosan had on the PMP of PEG-grafted chitosan. The results show that this PMP has a bell-shaped dependence on the buffer solution’s pH (optimal reaction pH was approximately 4). The pH-dependence was presumably due to two phenomena: (1) the increased reactivity of the PEG-grafted chitosan’s amino group coupled with the increase in pH of the buffer solution; and (2) the decreased conversion of the PEG’s acetal group into an aldehyde group coupled with the increased pH of the buffer solution. Figure 4c shows the effect that the reaction time of the Schiff base formation in the synthesis condition of the PEG-modified chitosan had on the PMP of PEG-grafted chitosan. The results suggested that the reaction time of Schiff base formation did not have a great influence on the PMP of PEG-grafted chitosan (1 h was the minimal requirement for the efficient synthesis of PEG-grafted chitosan). Figure 4d shows the effect that the reaction temperature of the Schiff base formation in the synthesis condition of the PEG-modified chitosan had on the PMP of PEG-grafted chitosan. The results show that there was no temperature dependence of the PMP of PEG-grafted chitosan (in Figure 4, we performed the subsequent reaction to reduce Schiff base formation for 18 h before the evaluation of PMP. Therefore, it was difficult to evaluate the reaction kinetics in the early stage correctly because the PEG-chitosan conjugation slightly proceeded during the subsequent reduction reaction).

### 3.3. Evaluation of the Solubility of PEG-Grafted Chitosan by Static Light Scattering Measurement

**Figure 5:**
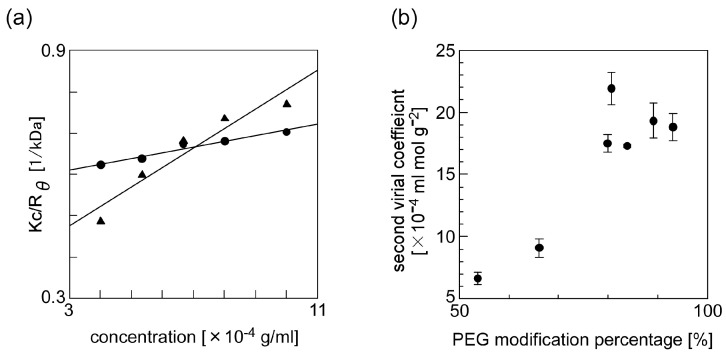
(**a**) The Debye plots of the PEG-grafted chitosans (the PMP of PEG-grafted chitosans was 53.7% (closed circle) and 80.7% (closed triangle)) and (**b**) the effect of the PMP of PEG-grafted chitosans on their second virial coefficient.

We used static light scattering measurement to evaluate the effect of the PMP of PEG-grafted chitosan on the solubility of the PEG-grafted chitosan. Under a low concentration condition, the scattering intensity of the analyte-containing sample solution can be described in reference to the Rayleigh equation *KC*/*R_θ_* = 2*A*_2_*C* + 1/*M*, where *K* is an optical constant, *R_θ_* is a Rayleigh ratio of the analyte intensity to an incident intensity, *A*_2_ is a second virial coefficient, and *M* is a molecular weight of the analyte [42,43,44]. In our experiments, we adjusted *C*, a concentration of the PEG-grafted chitosan, to within the range of 0.4 and 1.0 g/L. A plot of *KC*/*R_θ_* versus *C*, known as a Debye plot, is linear, with an intercept equivalent to 1/*M* and a slope that is proportional to the second virial coefficient. Figure 5a shows the typical Debye plots that we obtained from the results of static light scattering measurements of PEG-grafted chitosan. The results show that the slope for the PEG-grafted chitosan with a high PMP is higher than that for the PEG-grafted chitosan with a low PMP. A second virial coefficient can help analyze the solubility of molecules in a solvent, where a second virial coefficient of molecules increases as their solubility increases. The results in Figure 5a clearly show that there was a difference in the second virial coefficients of the PEG-grafted chitosans with different PMP; the results also show that PEG modification is effective for the increase of chitosan. Figure 5b summarizes the effect that the PMP of PEG-grafted chitosan had on the solubility of PEG-grafted chitosan in water: the results clearly show that the second virial coefficient of PEG-grafted chitosan increased as the PMP of PEG-grafted chitosan increased. These results suggest that chitosan, which is water insoluble, underwent an increase in its solubility after we modified PEG, which has high water solubility.

### 3.4. Evaluation of the Gelation Property of PEG-Grafted Chitosan and Formation of the Hydrogel Sheet

We evaluated the gelation properties of PEG-grafted chitosan by mixing two solutions containing either PEG-modified chitosans or a crosslinkable polymeric micelle. Polymeric micelles were used as a crosslinker. Since the critical micelle concentration (CMC) of the block copolymer was low (ca. 0.001 w/w %), it is expected that the micelles maintains their structure in the polymeric gel networks. The rheometer gives the storage modulus (G’) and loss modulus (G”) that characterize elastic and viscous characteristics of hydrogels, respectively. G’ is approximately equal to G*, which represents the shear stiffness of the hydrogel. The gelation time is defined as the time when the solution phase changes from sol (G’ < G”, liquid-like behavior) to gel (G’ > G”, solid-like behavior). The gel strength is defined as the value of G’ at 60 min after the start of a measurement. The hydrogel was formed according to the Schiff base formation reaction. Hydrogel was not obtained when only PEG-grafted chitosan was present, whereas hydrogel was obtained only when PEG-grafted chitosan was mixed with aldehyde-terminated polymeric micelle. The results showed that block copolymers would form micelles to crosslink PEG-grafted chitosan. The gelation time was within a second (the results were consistent with our previous results on aldehyde-terminated polymeric micelle-based hydrogel in our previous papers) [3,5,6,36].

**Figure 6:**
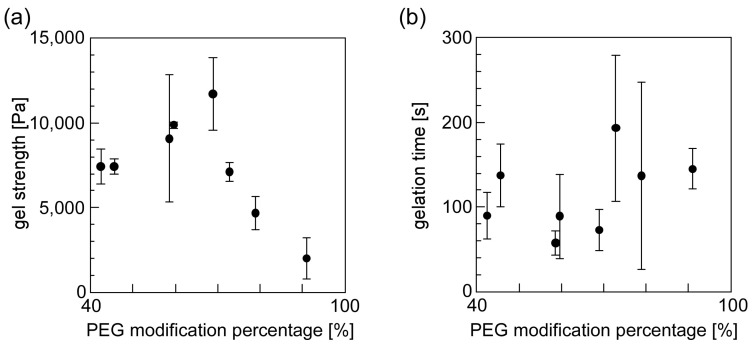
The effect of the PMP of PEG-grafted chitosans on their gelation properties ((**a**) gel strength and (**b**) gelation time).

Figure 6 shows the effect that the PMP of PEG-grafted chitosan had on its gelation properties. The gel strength increased, reached the saturation value, and decreased, as the PMP of PEG-grafted chitosan increased. The gelation time slightly lengthened as the PMP of PEG-grafted chitosan increased. Although the static light scattering measurement (Figure 5b) shows that the solubility of PEG-grafted chitosan to water increased as the PMP of PEG-grafted chitosan increased, the excess modification of PEG to chitosan resulted in a decrease in the amino groups of the chitosan backbone. Thus, in the high PMP range, an insufficient crosslink point was formed in the resulting hydrogel, and the gelation time lengthened. These results suggest that the optimal PMP was approximately 70% for forming hydrogel consisting of PEG-grafted chitosan and crosslinkable polymeric micelles.

**Figure 7:**
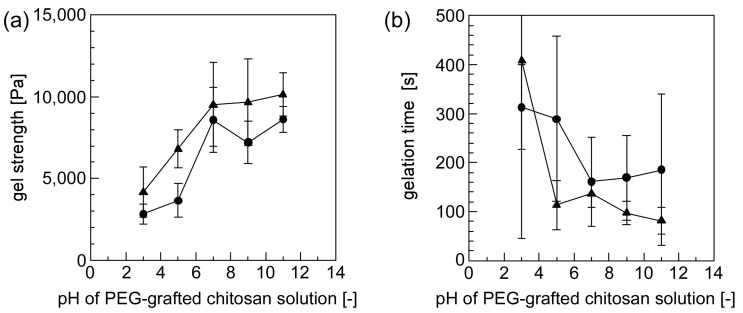
The effect of the pH of solutions containing either PEG-grafted chitosan or a crosslinkable polymeric micelle on their gelation properties ((**a**) gel strength and (**b**) gelation time). The pH of the polymeric micelle solution was 5 (closed circle) and 7 (closed triangle).

Figure 7 shows the effect that the pH attributable to solutions containing PEG-grafted chitosan had on its gelation properties. The hydrogel formed within a few minutes for all the combinations of PEG-grafted chitosan solutions (at pH 3–11) and polymeric micelles solutions (at pH 5 and 7). The gel strength increased and gelation time shortened, as the pH of the PEG-grafted chitosan solution increased. The degree of the protonation attributable to the amino groups present on the chitosan backbone is dependent on the pH of the PEG-grafted chitosan solutions. Because the amino groups of the chitosan backbone are protonated in a low pH region, the reactivity of amino groups increased as the pH of PEG-grafted chitosan solutions increased. When the pH of the PEG-grafted chitosan solutions was between 7 and 11, the gel strength was almost constant. This pattern suggests that the amino groups of the chitosan backbone were not influenced by protons in the pH range extending from 7 to 11. Furthermore, the gel strength was slightly higher and the gelation time was slightly shorter when the polymeric solution at pH 7 was used than when the polymeric solution at pH 5 was used. This contrast is presumably due to differences in the hydrogels’ inner pH environment: the result suggests that the gelation reaction is enhanced at higher pH.

**Figure 8:**
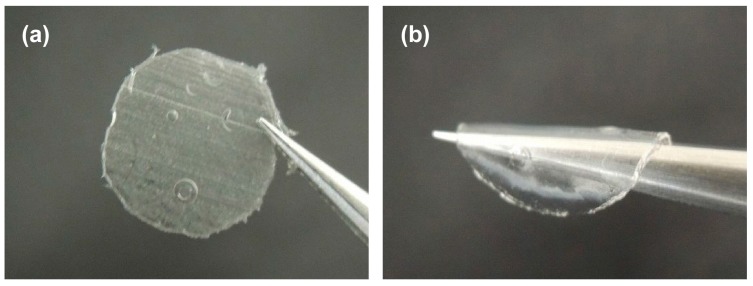
The sheet-shaped PEG-grafted chitosan hydrogel: (**a**) dried sheet-shaped gel; (**b**) flexible sheet-shaped gel after immersion in water.

Figure 8 shows a finally obtained hydrogel sheet from mixed solutions containing either PEG-modified chitosan or reactive polymeric micelles. The mixed solution was introduced at a gap of 1.0 mm between two plates made of acrylic acid resin. After three days, a sheet was obtained without any support medium (one can select the appropriate support medium for the clinical application). After the sheet completely dried, it was rigid and thin, as shown in Figure 8a. The dried rigid and thin sheet is expected to be suitable for stable preservation. After immersing the dried sheet in water, we successfully obtained a flexible hydrogel sheet, as shown in Figure 8b. The hydrogel can be reversibly dried and moistened without a collapse. The chitosan gel sheet showed optical transmittance of 86.0 ± 2.0% (*n* = 3), which was consistent with the values that were reported for chitosan-based sheets [45,46,47].

## 4. Conclusions

Sheets have several advantages for their biomedical applications. In particular, thin sheets have a large contact area relative to the drug-targeted site, leading to advantages over conventional particle-shaped drug carriers. We confirmed that various factors (i.e., the weight ratio of PEG/chitosan, the pH of the buffer solution, reaction times, and reaction temperatures) in the preparation stage of PEG-grafted chitosans affected the PMP of PEG-grafted chitosans. Finally, we succeeded in preparing a flexible hydrogel sheet that contained a drug carrier and has reversible dry and wet forms. Potential applications of our material would be wound-dressing biomaterials. Furthermore, excellent chitosan-based materials that can render passive dressings active dressing in a cost effective way were recently proposed [48]. One of the attractive applications of our gel sheets is such a bio-coating material for tissues that can also be used as wound gauzes.

Aldehyde micelles are safe because there is no risk of infectious contaminations. However, an excess amount of aldehyde groups makes the micelle toxic. Efforts are now being made towards controlling the aldehyde groups on the surface of the micelle. Furthermore, to fully understand the gelation of PEG-grafted chitosans, it is important to evaluate the thermodynamic compatibility of PEG-grafted chitosans and reactive cross-linkable polymeric micelles in terms of the Flory–Huggins interaction parameters in the future works. Although further studies on the optimization of factors related to the sheet’s preparation and drug release properties are necessary, the results obtained in this paper show that the incorporation of drug carriers into biomaterials is a novel approach to improve functionality.

## References

[1] Loo Y., Wong Y.-C., Cai E.Z., Ang C.-H., Raju A., Lakshmanan A., Koh A.G., Zhou H.J., Lim T.-C., Moochhala S.M. (2014). Ultrashort peptide nanofibrous hydrogels for the acceleration of healing of burn wounds. Biomaterials.

[2] Dong Y., Hassan W.U., Kennedy R., Greiser U., Pandit A., Garcia Y., Wang W. (2014). Performance of an in situ formed bioactive hydrogel dressing from a PEG-based hyperbranched multifunctional copolymer. Acta Biomater..

[3] Murakami Y., Yokoyama M., Okano T., Nishida H., Tomizawa Y., Endo M., Kurosawa H. (2007). A novel synthetic tissue-adhesive hydrogel using a crosslinkable polymeric micelle. J. Biomed. Mater. Res..

[4] Murakami Y., Yokoyama M., Nishida H., Tomizawa Y., Kurosawa H. (2008). A simple hemostasis model for the quantitative evaluation of hydrogel-based local hemostatic biomaterials on tissue surface. Colloids Surf. B Biointerfaces.

[5] Murakami Y., Yokoyama M., Nishida H., Tomizawa Y., Kurosawa H. (2009). In vivo and in vitro evaluation of gelation and hemostatic properties of a novel tissue-adhesive hydrogel containing a cross-linkable polymeric micelle. J. Biomed. Mater. Res. B Appl. Biomater..

[6] Uchida Y., Fukuda K., Murakami Y. (2013). The hydrogel containing a novel vesicle-like soft crosslinker, a “trilayered” polymeric micelle, shows characteristic rheological properties. J. Polym. Sci. B Polym. Phys..

[7] Murata M., Uchida Y., Takami T., Ito T., Anzai R., Sonotaki S., Murakami Y. (2017). Dual drug release from hydrogels covalently containing polymeric micelles that possess different drug release properties. Colloids Surf. B Biointerfaces.

[8] Moroishi H., Yoshida C., Murakami Y. (2013). A free-standing, sheet-shaped, “hydrophobic” biomaterial containing polymeric micelles formed from poly(ethylene glycol)-poly(lactic acid) block copolymer for possible incorporation/release of “hydrophilic” compounds. Colloids Surf. B Biointerfaces.

[9] Anzai R., Murakami Y. (2015). Poly(ε-caprolactone) (PCL)-polymeric micelle hybrid sheets for the incorporation and release of hydrophilic compounds. Colloids Surf. B Biointerfaces.

[10] Anzai R., Takami T., Uchida Y., Murakami Y. (2017). Poly(ε-caprolactone) (PCL) hybrid sheets containing polymeric micelles: Effects of inner structures on the material properties of the sheet. Mater. Sci. Eng. C.

[11] Kanakubo Y., Ito F., Murakami Y. (2010). Novel one-pot facile technique for preparing nanoparticles modified with hydrophilic polymers on the surface via block polymer-assisted emulsification/evaporation process. Colloids Surf. B Biointerfaces.

[12] Takami T., Murakami Y. (2011). Development of PEG-PLA/PLGA microparticles for pulmonary drug delivery prepared by a novel emulsification technique assisted with amphiphilic block copolymers. Colloids Surf. B Biointerfaces.

[13] Takami T., Murakami Y. (2014). Unexpected and successful “one-step” formation of porous polymeric particles only by mixing organic solvent and water under “low-energy-input” conditions. Langmuir.

[14] Yoneki N., Takami T., Ito T., Anzai R., Fukuda K., Kinoshita K., Sonotaki S., Murakami Y. (2015). One-pot facile preparation of PEG-modified PLGA nanoparticles: Effects of PEG and PLGA on release properties of the particles. Colloids Surf. A Physicochem. Eng. Asp..

[15] Nishimura S., Takami T., Murakami Y. (2017). Porous PLGA microparticles formed by “one-step” emulsification forpulmonary drug delivery: The surface morphology and theaerodynamic properties. Colloids Surf. B Biointerfaces.

[16] Nishihara M., Murakami Y., Shinoda T., Yamamoto J., Yokoyama M. (2008). Synthesis and characterization of a temperature-responsive amphiphilic block copolymer containing a liquid crystalline unit. Chem. Lett..

[17] Gupta R., Shea J., Scafe C., Shurlygina A., Rapoport N. (2015). Polymeric micelles and nanoemulsions as drug carriers: Therapeutic efficacy, toxicity, and drug resistance. J. Control. Release.

[18] Emami J., Rezazadeh M., Hasanzadeh F., Sadeghi H., Mostafavi A., Minaiyan M., Rostami M., Davies N. (2015). Development and in vitro/in vivo evaluation of a novel targeted polymeric micelle for delivery of paclitaxel. Int. J. Biol. Macromol..

[19] Chen Y., Zhang W., Huang Y., Gao F., Sha X., Fang X. (2015). Pluronic-based functional polymeric mixed micelles for co-delivery of doxorubicin and paclitaxel to multidrug resistant tumor. Int. J. Pharm..

[20] Cabral H., Kataoka K. (2014). Progress of drug-loaded polymeric micelles into clinical studies. J. Control. Release.

[21] Lu Y., Park K. (2013). Polymeric micelles and alternative nanonized delivery vehicles for poorly soluble drugs. Int. J. Pharm..

[22] Uchida Y., Murakami Y. (2010). Trilayered polymeric micelle: A newly developed macromolecular assembly that can incorporate hydrophilic compounds. Colloids Surf. B Biointerfaces.

[23] Uchida Y., Murakami Y. (2011). Successful preferential formation of a novel macromolecular assembly–trilayered polymeric micelle—That can incorporate hydrophilic compounds: The optimization of factors affecting the micelle formation from amphiphilic block copolymers. Colloids Surf. B Biointerfaces.

[24] Pillai C.K.S., Paul W., Sharma C.P. (2009). Chitin and chitosan polymers: Chemistry, solubility and fiber formation. Prog. Polym. Sci..

[25] Boucard N., Viton C., Agay D., Mari E., Roger T., Chancerelle Y., Domard A. (2007). The use of physical hydrogels of chitosan for skin regeneration following third-degree burns. Biomaterials.

[26] Murakami K., Aoki H., Nakamura S., Nakamura S.I., Takikawa M., Hanzawa M., Kishimoto S., Hattori H., Tanaka Y., Kiyosawa T. (2010). Hydrogel blends of chitin/chitosan, fucoidan and alginate as healing-impaired wound dressings. Biomaterials.

[27] Sugimoto M., Morimoto M., Sashiwa H., Saimoto H., Shigemasa Y. (1998). Synthesis and bioactivities of poly (ethylene glycol)-chitosan hybrids. Carbohydr. Polym..

[28] Shantha K.L., Harding D.R.K. (2002). Synthesis and characterisation of chemically modified chitosan microspheres. Carbohydr. Polym..

[29] Zhang Q., Wang C.R., Babukutty Y., Ohyama T., Kogoma M., Kodama M. (2002). Biocompatibility evaluation of ePTFE membrane modified with PEG in atmospheric pressure glow discharge. J. Biomed. Mater. Res..

[30] Lee H.J., Lee J.S., Chansakul T., Yu C., Elisseeff J.H., Yu S.M. (2006). Collagen mimetic peptide-conjugated photopolymerizable PEG hydrogel. Biomaterials.

[31] Murakami Y., Hirata A. (1999). Complex between α-chymotrypsin and poly(ethylene glycol) catalytically active in organic media. Biotechnol. Tech..

[32] Murakami Y., Hirata A. (1999). Poly(ethylene glycol)-α-chymotrypsin complex catalytically active in anhydrous isooctane. J. Biosci. Bioeng..

[33] Murakami Y., Hoshi R., Hirata A. (2001). Borate buffer dramatically enhances the activity of poly(ethylene glycol)-α-chymotrypsin complex catalytically active in anhydrous isooctane than conventional phosphate buffer even at low concentration. Biotechnol. Lett..

[34] Murakami Y., Hoshi R., Hirata A. (2003). Characterization of polymer–enzyme complex as a novel biocatalyst for nonaqueous enzymology. J. Mol. Catal. B Enzym..

[35] Murakami Y., Hirata A. (1998). Enzymatic synthesis of peptides-review. Seibutsu Kogaku Kais.

[36] Ito T., Yoshida C., Murakami Y. (2013). Design of novel sheet-shaped chitosan hydrogel for wound healing: A hybrid biomaterial consisting of both PEG-grafted chitosan and crosslinkable polymeric micelles acting as drug containers. Mater. Sci. Eng. C.

[37] Contardi M., Heredia-Guerrero J.A., Perotto G., Valentini P., Pompa P.P., Spanò R., Goldoni L., Bertorelli R., Athanassiou A., Bayer I.S. (2017). Transparent ciprofloxacin-povidone antibiotic films and nanofiber mats as potential skin and wound care dressings. Eur. J. Pharm. Sci..

[38] Kathuria N., Tripathi A., Kar K.K., Kumar A. (2009). Synthesis and characterization of elastic and macroporous chitosan-gelatin cryogels for tissue engineering. Acta Biomater..

[39] El-Sherbiny I.M., Smyth H.D.C. (2010). Poly(ethylene glycol)–carboxymethyl chitosan-based pH-responsive hydrogels: Photo-induced synthesis, characterization, swelling, and in vitro evaluation as potential drug carriers. Carbohydr. Res..

[40] Bhattarai N., Ramay H.R., Gunn J., Matsen F.A., Zhang M. (2005). PEG-grafted chitosan as an injectable thermosensitive hydrogel for sustained protein release. J. Control. Release.

[41] Hu Y., Jiang H., Xu C., Wang Y., Zhu K. (2005). Preparation and characterization of poly(ethylene glycol)-g-chitosan with water- and organosolubility. Carbohydr. Polym..

[42] Zimm B.H. (1948). The Scattering of light and the radial distribution function of high polymer solutions. J. Chem. Phys..

[43] Vekilov P.G., Feeling-Taylor A.R., Petsev D.N., Galkin O., Nagel R.L., Hirsch R.E. (2002). Intermolecular interactions, nucleation, and thermodynamics of crystallization of hemoglobin C. Biophys. J..

[44] Debye P. (1947). Molecular-weight determination by light scattering. J. Phys. Colloid Chem..

[45] Liu J., Liu S., Wu Q., Gu Y., Kan J., Jin C. (2017). Effect of protocatechuic acid incorporation on the physical, mechanical, structural and antioxidant properties of chitosan film. Food Hydrocoll..

[46] Chen H., Hu X., Chen E., Wu S., McClements D.J., Liu S., Li B., Li Y. (2016). Preparation, characterization, and properties of chitosan films with cinnamaldehyde nanoemulsions. Food Hydrocoll..

[47] Soni B., Hassan E.B., Schilling M.W., Mahmound B. (2016). Transparent bionanocomposite films based on chitosan and TEMPO-oxidized cellulose nanofibers with enhanced mechanical andbarrier properties. Carbohydr. Polym..

[48] Romano I., Ayadi F., Rizzello L., Summa M., Bertorelli R., Pompa P.P., Brandi F., Bayer I.S., Athanassiou A. (2015). Controlled antiseptic/eosin release from chitosan-based hydrogel modified fibrous substrates. Carbohydr. Polym..

